# Leveraging Reddit for Suicidal Ideation Detection: A Review of Machine Learning and Natural Language Processing Techniques

**DOI:** 10.3390/ijerph191610347

**Published:** 2022-08-19

**Authors:** Eldar Yeskuatov, Sook-Ling Chua, Lee Kien Foo

**Affiliations:** Faculty of Computing and Informatics, Multimedia University, Persiaran Multimedia, Cyberjaya 63100, Malaysia

**Keywords:** suicidal ideation detection, machine learning, natural language processing, text mining

## Abstract

Suicide is a major public-health problem that exists in virtually every part of the world. Hundreds of thousands of people commit suicide every year. The early detection of suicidal ideation is critical for suicide prevention. However, there are challenges associated with conventional suicide-risk screening methods. At the same time, individuals contemplating suicide are increasingly turning to social media and online forums, such as Reddit, to express their feelings and share their struggles with suicidal thoughts. This prompted research that applies machine learning and natural language processing techniques to detect suicidality among social media and forum users. The objective of this paper is to investigate methods employed to detect suicidal ideations on the Reddit forum. To achieve this objective, we conducted a literature review of the recent articles detailing machine learning and natural language processing techniques applied to Reddit data to detect the presence of suicidal ideations. Following the Preferred Reporting Items for Systematic Reviews and Meta-Analyses guidelines, we selected 26 recent studies, published between 2018 and 2022. The findings of the review outline the prevalent methods of data collection, data annotation, data preprocessing, feature engineering, model development, and evaluation. Furthermore, we present several Reddit-based datasets utilized to construct suicidal ideation detection models. Finally, we conclude by discussing the current limitations and future directions in the research of suicidal ideation detection.

## 1. Introduction

Suicide is a global public-health problem. According to the World Health Organization, approximately 703,000 people commit suicide every year [1]. It is the world’s fourth leading cause of death among young people aged 15 to 29 years old. Moreover, it is estimated that there are more than 20 attempts for every completed suicide [2].

The causes of suicide are largely complicated and result from the interaction of multiple factors that can be grouped into three categories: health factors, environmental factors, and factors related to personal history, such as childhood abuse or previous suicide attempts [3,4]. Other examples of suicide risk factors include mental disorder, physical illness, substance abuse, domestic violence, bullying, relationship problems, and other stressful life events. Due to the complexity of the problem, no single risk factor can be reliably used to predict suicide [5]. For instance, despite the strong association between suicide and depression, a depression diagnosis alone has a limited ability to predict suicide. More recently, the issue of suicide has been further exacerbated by the impact of the COVID-19 pandemic [6]. In particular, social isolation—which resulted from measures imposed to curb the spread of the virus—was linked to increased suicide risk.

People with suicide risk fall into two classes: ideators and attempters [7]. Suicidal ideation is a broad term that describes thoughts and behaviors ranging from being preoccupied with death to planning a suicide attempt [8]. The suicidal ideations can be passive and active. Passive suicidal ideation involves thinking about suicide and wishing to be dead, whereas active suicidal ideation implies intending and planning an attempt to take one’s own life [8]. While it is believed that passive suicidal ideation poses a lower risk, both types need to be carefully assessed by mental health professionals, since passive suicidal ideation can rapidly transform into the active form [9]. This can happen when a person’s circumstances or health condition worsen.

The early detection of suicidal ideation expressed by an at-risk individual is key to effective prevention, as it facilitates timely intervention by mental health professionals [10]. However, there are several challenges associated with suicide prevention. They include (1) social stigma, (2) limited access to professional help, and (3) inadequate training of clinicians in dealing with suicidal patients [11]. The combination of these factors creates a new challenge—(4) fragmented professional care, which entails having large time gaps between mental health assessments [11].

At the same time, an increasing number of at-risk individuals are turning to online communication channels to express their feelings and discuss their suicidal thoughts [12,13,14]. This tendency prompted research that focuses on detecting suicide risk and other mental health issues on social networks and online forums by applying machine learning (ML) and natural language processing (NLP) techniques [10,13,15]. The quantifiable signals in user-generated online data aid researchers in gaining insight into an individual’s emotional state and detecting cues indicative of suicidality [16,17]. The feasibility of such an approach has been demonstrated by numerous studies on different mental health disorders. For examples, the authors of [18] used the textual data from Facebook posts of consenting study participants to predict depression diagnoses recorded in their electronic medical records with high accuracy, using a logistic regression model. In the study of [19], using pre-trained machine learning models, the researchers detected negative changes in Twitter users’ sentiment, stress, anxiety, and loneliness measures after the declaration of emergency in the US due to the COVID-19 pandemic.

Over the past five years, there have been several literature reviews and surveys that investigated the application of ML techniques to analyze mental health disorders and suicidality.

Chancellor and De Choudhury [20] conducted a systematic review of 75 studies to evaluate the state of the art in detecting mental health issues on social media. The authors sourced papers from the ACM Digital Library, Google Scholar, and Web of Science. The examined studies focused on a wide range of disorders and symptomatology, including depression, suicidality, eating disorders, anxiety, bipolar disorder, post-traumatic stress disorder, and others. Multiple social media platforms were analyzed by reviewed studies, including Twitter, Reddit, Weibo, and Facebook. The results of the review outlined methods of data annotation, data quality management, feature engineering, algorithm selection, and validation. The authors also discussed concerns over construct validity and proposed better reporting practices to facilitate the reproducibility of research.

Skaik and Inkpen [21] investigated the use of social media for mental health surveillance. Their review presented the trends and tools used in the field, as well as different data collection methods, including questionnaires, forums, and social media posts. The authors followed the Preferred Reporting Items for Systematic Reviews and Meta-Analyses (PRISMA) guidelines to select 110 publications from six sources, namely PubMed (Bethesda, MD, USA), ACM Digital Library (New York, NY, USA), Springer (Berlin/Heidelberg, Germany), Elsevier (Amsterdam, The Netherlands), IEEE Xplore (New York, NY, USA), and Google Scholar (Mountain View, CA, USA). The studies included in the review focused on identifying depression, post-traumatic stress disorder, and suicidality on various platforms, such as Twitter (San Francisco, CA, USA), Weibo (Beijing, China), and Reddit (San Francisco, CA, USA). The authors also discussed the application of ML and NLP techniques for population-level mental health surveillance.

Castillo-Sánchez et al. [6] conducted a scoping review of 16 studies to identify ML techniques used to predict suicide risk on social networks. The papers were searched from PubMed, ScienceDirect, IEEE Xplore, and Web of Science and selected for review using the PRISMA methodology. The majority of the studies included in the review focused on Twitter. The authors provided descriptions of the reviewed studies and reported on the data sources and model development steps. They concluded by discussing common issues found in the corpus.

Ji et al. [10] provided a review of ML methods used for suicidal ideation detection and discussed their applications in various domains, including questionnaires, electronic health records, suicide notes, and user-generated online content. The authors examined how studies approached content analysis and feature engineering. They also explored deep learning methods and provided a list of datasets used in the research area.

Meanwhile, ref. [22] reviewed eight studies to highlight the feature extraction methods and classifier algorithms used for suicidal ideation prediction. The authors summarized the available datasets constructed from Twitter, Reddit, Vkontakte (Saint Petersburg, Russia), and Tumblr (New York, NY, USA) data, as well as datasets consisting of interview and questionnaire responses.

While there are reviews investigating ML techniques utilized for identifying mental health issues, and specifically suicidality, their scope covers a broad range of mental health symptomatology and includes sources of data other than social media, such as questionnaires, electronic health records, and suicide notes. Moreover, the literature reviews investigating ML techniques applied to social networks tend to focus on Twitter as a source of data. For instance, 40% of studies reviewed by [20] were conducted on Twitter. Similarly, 36% of studies reviewed by [21] used Twitter as a platform of choice. Although there are some similarities in the methodologies used for detecting suicidal ideations on different social networks, the detection of suicidal ideation on Reddit has certain distinctions due to characteristics specific to the platform.

Our aim in this paper is to explore the methods used for detecting suicidal ideations on Reddit specifically. There are other review studies for suicidal ideation detection using other social media data, such as Twitter and Facebook (Menlo Park, CA, USA) [6,20]. These reviews, however, are merely focusing on the data preprocessing steps and the machine learning methods used to detect suicidal ideation. Our paper provides a comprehensive review of the entire process from data collection and annotation, data preprocessing, feature engineering to model development and model validation. To the best of our knowledge, this is the first review article that primarily focuses on Reddit as an avenue of research for studying suicidal ideation detection. The main contributions are as follows:

- We present the state of the art in suicidal ideation detection by reviewing the prevalent methods within these rational aspects:
  1. How do current studies approach data collection and annotation?
  2. What techniques are used to extract suicide-indicative features?
  3. What algorithms are used for detecting suicidal ideations?
- We provide descriptions of several Reddit-based datasets used in the domain;
- We discuss current limitations and future directions of the research in detecting suicidal ideation.

The remainder of this review paper is structured as follows: Section 2 discusses the methodology in carrying out the literature search; Section 3 presents the findings and outlines the common techniques used by the studies; and the limitations and the future directions of the research are discussed in Section 4.

## 2. Methodology

In this section, we detail how the literature search was carried out.

### 2.1. Search Strategy

To investigate the methods used for suicidal ideation detection on Reddit, we conducted a literature review of the studies published between 2018 and 2022 using the Preferred Reporting Items for Systematic Reviews and Meta-Analyses (PRISMA) guidelines [23]. The papers were searched from PubMed, IEEE Xplore, ScienceDirect, and Google Scholar databases. The online search took place from January 2022 to May 2022. The following search terms were used: “detect” OR “predict” AND “suicidal ideation” OR “suicidality” OR “suicide risk” AND “social media” OR “forum” OR “Reddit” AND “machine learning” OR “deep learning” OR “natural language processing”. The reason we included both “social media” and “forum” in the search terms is that some researchers categorize Reddit as social media whereas others categorize it as a forum. Therefore, if we only included “forum” in our search terms we would risk overlooking potentially relevant articles. In addition, we examined the references sections of included publications to identify additional sources.

### 2.2. Eligibility Criteria

We limited the literature type to journal articles and proceedings from conferences and workshops. To be included in the review the following inclusion criteria had to be satisfied: (1) published between 2018 and 2022; (2) an original study; (3a) apply ML and NLP techniques to detect suicidal ideations; or (3b) apply ML and NLP to determine the level of suicide risk; (4) use Reddit data source; and (5) focus on suicide risk and suicidal ideations. We limited the search to papers published in the past five years as we wanted to explore recent techniques.

To further narrow down the corpus, the following exclusion criteria were used: (1) studies examining other mental disorders; (2) review papers; (3) studies that focused only on feature extraction and did not conduct a suicidal ideation prediction; and (4) studies that used social media platforms other than Reddit.

### 2.3. Selection Process

During the initial search, we identified 121 studies: 110 studies from search databases and 11 studies from citations. Out of 110 studies, 15 studies were obtained from IEEE Xplore, 3 studies from PubMed, 8 studies from ScienceDirect, and the remaining 84 studies were obtained from Google Scholar. In the first stage, 6 studies were removed due to duplication. Having analyzed the titles and abstracts, we removed 11 papers because they were literature reviews and another 22 studies were removed because they were published before 2018.

**Figure 1:**
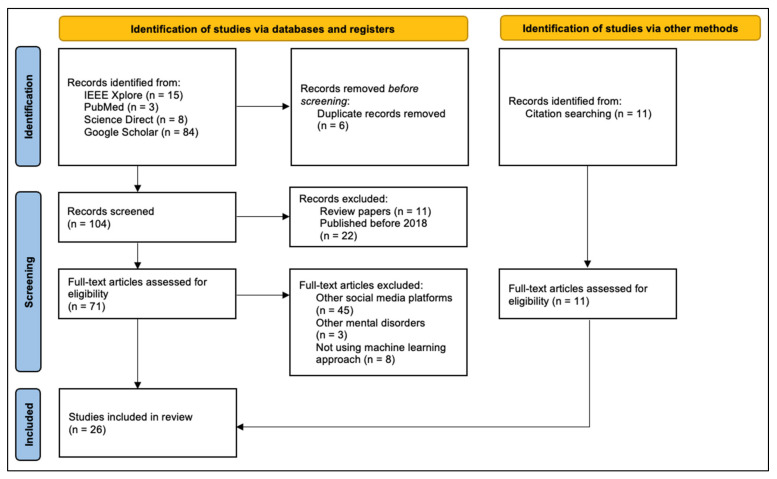
Study selection flowchart based on PRISMA guidelines.

After reading full-text articles, we excluded a total of 56 studies: 45 studies, which focused on other social media platforms; 3 studies, which focused on other mental health issues; and 8 studies used approaches other than ML. As a result, 26 studies were included in the final review. Figure 1 illustrates the PRISMA flowchart diagram representing the study selection process.

## 3. Results

In this section, the findings of the review are analyzed and synthesized to provide the answers to the posed research questions, which were defined to uncover the methodology used in the domain. We start by exploring the rationale of detecting suicidal ideations on Reddit and then we investigate specific ML and NLP techniques applied for that purpose.

### 3.1. Detection of Suicidal Ideation on Social Media

The outcomes of the research in this area can help address the existing challenges in suicide prevention. The social stigma related to having suicidal ideations has a particularly significant effect. The fear of social stigma has been shown to discourage individuals at risk of suicide from discussing their experiences in person and seeking support [22,24,25,26]. Further, it obstructs the extant suicide-risk screening methods, such as questionnaires and interviews, since they require patients to explicitly disclose their intentions to commit suicide [27]. According to a meta-analysis of 71 studies, on average, nearly 80% of people in non-psychiatric settings—primary healthcare patients, general population, military personnel, and incarcerated individuals—who died by suicide did not reveal their suicidal intentions when they were surveyed before their suicide attempt [28]. Thus, there is a need for novel suicidality detection methods that do not require face-to-face interactions [24]. In this case, detecting suicidal ideations on online platforms can be more effective since the anonymity of social media and forums enables people to openly share their struggles with suicidal thoughts without fear of judgment [11,16,29,30].

Although the Columbia-Suicide Severity Rating Scale (C-SSRS) has been widely used as a screening instrument, the administration of C-SSRS may place a burden on health-care providers [31]. Therefore, another motivation for detecting suicidal ideations on online platforms is to reduce the load on the health-care system. The goal is to create a tool that would automatically and instantaneously detect if a user is exhibiting any signs of suicidality based on their online activity before engagement with providers. Ideally, these screening tools should be highly scalable and adaptable so that they can be used with a variety of data sources and be readily integrated into existing health-care IT systems [10,31]. The adoption of such suicidal ideation detection tools can assist mental health professionals and even those without specialized training (e.g., primary-care physicians and social workers) in quickly identifying individuals at risk and making informed decisions [26].

Studying the online activity for suicidal ideation detection can also help address the challenges of fragmented care for existing patients [32]. Given that about 70% of psychiatric patients are active on social media, mental health professionals can monitor their online activity to obtain information relevant to patients’ mental state during gaps in patient–clinician interactions [11]. In this scenario, suicidality detection tools can be employed to automatically detect signals of deteriorating mental condition and alert health-care providers, prompting them to attend to a patient under their care [31].

### 3.2. Reddit as a Source for Suicidal Ideation Detection

To detect suicidal ideations, researchers have studied different social media sites, such as Twitter, Facebook, and Reddit, as well as specialized support forums, such as ReachOut [6,10]. There is also a growing number of studies that investigate the Chinese microblogging website Weibo for detecting suicidality [6]. Out of these platforms, Reddit has generated particular interest among researchers due to its distinctive characteristics. Reddit is a popular online forum, covering a wide range of topics, with subcommunities called subreddits [21]. Currently, there are over 13 billion posts and comments distributed across more than 100,000 active communities [33]. More than 50 million active unique users interact with the platform in a single day. Researchers choose Reddit over other platforms as the source of data for several reasons.

Reddit posts have a higher character limit of 40,000 characters compared to Twitter, which only allows 280 characters [25]. It gives users more space to express their suicidal thoughts and describe their emotional state in more detail. The large posts provide a better insight into the author’s mental state [26]. By analyzing long passages of text, the researchers capture and extract textual features that sufficiently indicate suicidal ideations [10,27].

Reddit facilitates better anonymity [25,26]. As per Reddit’s privacy policy, users are not required to provide any identifying personal information or email address when creating an account [34]. The platform only requires a username and a password and the former does not have to relate to an actual name. This is unlike other social media sites. For instance, Facebook requires either a phone number or an email address during sign up, in addition to implementing a real-name policy that necessitates users to specify their real names on profiles [35]. Reddit users normally do not include their names and choose non-identifying ambiguous usernames. This level of anonymity allows people at risk of suicide to express themselves in an uninhibited fashion, without fear of social stigma [10,26,27]. This is valuable for researchers since unconstrained expounding of one’s experiences and feelings builds a genuine picture of the user’s psychological state.

Reddit has numerous specialized support forums dedicated to various mental health topics [26]. For example, the r/SuicideWatch subreddit is a subcommunity of 366,000 members where people share their suicidal thoughts, seek help, and provide support to others dealing with suicidal ideations [36,37]. This subreddit is extensively used by researchers as a source of suicidal posts to serve as positive samples in their datasets [10]. What further supports the validity of r/SuicideWatch as a source of genuine suicide-related posts is that this subreddit is monitored by moderators [25]. The moderators remove any irrelevant posts and posts that violate the community rules, e.g., abuse, criticism, and spam [37].

### 3.3. Machine Learning Approach for Suicidal Ideation Detection

The majority of the research, in general, approached the task of suicidal ideation detection as a classification problem where a predictive model was trained using ML techniques. Seven studies in the corpus performed a binary classification where the forum posts were classified as either containing suicidal ideations or not. Out of 26 studies, 19 performed a multiclass classification scheme where the examples were categorized into varying suicide risk levels, e.g., no risk, low risk, moderate risk, and severe risk. We also found that 18 studies aimed to make final predictions at the user level (i.e., detecting suicidal ideation in users, not in separate posts), whereas the remaining 8 studies made predictions at the post level. In the former scenario, all posts from one user were aggregated into a single document, which was later used to make a final prediction about the user’s suicidal ideation or risk level.

To build an accurate suicidal ideation detection model, it is essential to understand the key predictors of suicidality. The researchers utilized different NLP techniques to identify features that indicated suicidal ideations in a process called feature engineering.

Our findings show that the studies followed a similar model development framework. The framework components include data collection, data annotation, data preprocessing, feature engineering, model development, during which the classifier algorithms are trained, and model evaluation. Further in this section, the individual elements are discussed in more detail.

### 3.4. Data Collection

The first step in the process of building a classifier is obtaining a dataset containing sufficient posts for each class label. Having an accurate dataset with labeled examples is critical for the success of the ML model. The dataset is used to train and then test the model. The model’s predictive performance and its generalizability strongly depend on the quality and amount of training data. We identified two broad data collection approaches adopted by the studies: collecting data directly from Reddit and using datasets created by other researchers.

#### 3.4.1. Extracting Data from Reddit

Nine studies manually created their datasets by extracting public posts from Reddit. For example, ref. [7] used Google Cloud BigQuery to create a dataset consisting of 508,398 posts from 2008 to 2016, out of which 785 posts were annotated in two categories. The dataset contains posts from several subreddits, namely r/SuicideWatch, r/Depression, r/Anxiety, and r/ShowerThoughts. Ji et al. [13] created a dataset of 3549 posts containing suicidal ideations sourced from the r/SuicideWatch subreddit and balanced it with other 3652 posts not related to suicide from other popular subreddits, r/All, and r/Popular. Gaur et al. [26] developed a Reddit C-SSRS Suicide Dataset which consists of 500 users annotated in five different classes: supportive, suicide indicator, suicidal ideation, suicidal behavior, and suicide attempt. Shing et al. [38] created a UMD Reddit Suicidality Dataset consisting of 11,129 users (1,556,194 posts) who posted to the r/SuicideWatch subreddit. The same number of users who did not post in r/SuicideWatch was used as the control. The posts cover a period from 2006 to 2015. Yeo et al. [25] collected data from Reddit using Pushshift API and Python Reddit API Wrapper. The posts’ timeline spanned from June 2017 to June 2018. The posts were extracted from r/SuicideWatch and opioid-related subreddits. Similarly, ref. [30] used Reddit API to collect 2678 suicidal posts from r/SuicideWatch. Nikhileswar et al. [39] used Pushshift API to construct a balanced dataset of 232,074 posts. The authors sourced 116,037 suicidal posts from r/SuicideWatch and an equal number of posts were collected from r/Teenagers to represent non-suicidal posts.

#### 3.4.2. Using Available Datasets

The remaining 17 studies used available datasets created by other researchers. The UMD Reddit Suicidality Dataset, created by [38], was used by 12 studies that participated in the 2019 Computational Linguistics and Clinical Psychology Workshop (CLPsych 2019) [32]. Although not a CLPsych participant, ref. [40] also used this dataset in their research as it is available to non-participants upon approval from the authors. The authors of [16,41,42] used a dataset created by [13]. Kumar et al. [43] used the Reddit C-SSRS Suicide Dataset v1 developed by [26].

Table 1 provides a summary of datasets used in the included studies. The post-level dataset is used to detect suicidal ideation in users’ posts, while the user-level dataset is used to detect suicidal ideation in users.

### 3.5. Data Annotation

Supervised ML algorithms require annotated datasets. During the training stage, the algorithm generates a function that maps the relationship between the features and the target variables. To train the model to detect posts with suicidal ideations, the researchers need examples of posts annotated as suicidal and not suicidal. For the multiclass classification problem, posts with annotations for different suicide risk levels are required. From the reviewed papers, we identified three methods of annotation for the presence of suicidal ideations in the users’ posts.

#### 3.5.1. Expert Annotations

The first method of annotation enlisted the help of domain experts—clinical psychiatrists and psychologists—to annotate the examples. Four of the datasets used in the corpus were annotated by experts. For example, ref. [26] involved four practicing psychiatrists to annotate the subset of 500 users, consisting of 15,755 posts, when developing a Reddit C-SSRS Suicide Dataset. The annotation scheme was based on the C-SSRS questionnaire. Each of the experts annotated the same set of posts and then the authors measured the agreement level between annotators using Krippendorff’s α metric. Aladağ et al. [7] engaged two psychiatrists to annotate 175 posts from the r/SuicideWatch subreddit, while the author annotated 610 posts from other subreddits under their professional consultancy. Four mental health experts volunteered to annotate the subset of the UMD Reddit Suicidality Dataset (245 users) into four levels of suicide risk: no risk, low risk, moderate risk, and severe risk [38]. Krippendorff’s α metric was used to assess inter-annotator agreement. The researchers found that the agreement among annotators increased if more detailed instructions were provided to them.

#### 3.5.2. Crowdsourced Annotations

Most of the studies in the corpus used a dataset that was annotated through crowdsourcing. Yao et al. [25] engaged eligible Amazon Mechanical Turk workers to annotate 500 posts from the r/Opiates subreddit and 500 posts from the r/SuicideWatch subreddit using instructions based on the “Clues to Suicide” book, written by Edwin Shneidman, the founder of the American Association of Suicidology. The r/Opiates posts were labeled as either “Yes, implies opioid addiction” or “No opioid addiction”, whereas r/SuicideWatch posts were labeled as “Yes, risk of suicide” or “No risk of suicide.” Similarly, a subset of 1242 users from the UMD Reddit Suicidality Dataset was annotated by crowdsource workers [38]. The researchers provided the CrowdFlower platform workers with the annotation instructions. This subset was used in CLPsych 2019 Shared Task as the organizers argued it would be easier to repeat the task with crowdsourced annotations rather than expert ones.

#### 3.5.3. Community Affiliation

Another method for obtaining annotations is relying on community affiliation. This approach is based on the assumption that the posts made on suicide forums contain suicidal ideations. Normally, studies employ such an approach to source posts potentially containing suicidal ideations for further manual annotation [7,25]. However, the authors of [13,39,44] relied solely on community affiliation as the source of labels. For instance, [13] created a dataset where 3549 posts from r/SuicideWatch were labeled as suicidal and 3652 posts from r/All and r/Popular subreddits were used as non-suicidal examples. Similarly, ref. [39] used 116,037 r/SuicideWatch posts to represent suicidal posts and an equal number of posts from r/Teenager as non-suicidal posts. In other words, all posts from the r/SuicideWatch subreddit were assumed and directly annotated as containing suicidal ideations, whereas the content from other non-mental-health subreddits was used as a control that represented negative samples.

### 3.6. Data Preprocessing

The data collected from Reddit consist of raw, unstructured text and contain noise that can negatively impact the predictive performance of the model. The noise includes punctuation, special characters, URLs, emails, etc. The raw text needs to be converted into a numerical representation before it can be fed into a classifier. During the preprocessing stage, the input data are cleaned and standardized. Therefore, it is an important step that lays the foundation for feature extraction and classification. In this section, the most common preprocessing techniques are outlined.

#### 3.6.1. Data Cleaning

The data cleaning steps were taken by the studies to remove duplicate records and elements that do not carry semantic meaning, such as URLs, emails, special characters, newline symbols, HTML tags, and punctuation. For example, ref. [39] used Python’s built-in regular expression package to perform data cleaning. While [42,45] removed emojis from the data, ref. [38] converted them into corresponding text. Seven studies chose to remove stop words because they occur frequently in text and have little semantic importance. In contrast, ref. [46] kept them in the data as they argued that pronouns, articles, and prepositions can contain clues about users’ emotional state, personality, and connection to other people.

#### 3.6.2. Tokenization

Tokenization is a key preprocessing step that must be applied to text. It is a process of breaking down the passage of text into a list of discrete units called tokens [47]. The size of tokens determines the level of granularity at which the textual data are analyzed and processed. For instance, if the tokenization is conducted at a sentence level, one sentence is treated as one token. Similarly, if word tokenization is applied, then each word in the string represents a separate token. The NLTK Python package was used by [16,40,48] to perform tokenization, whereas [38,49] chose the SpaCy package to split the text into individual tokens. Ríssola et al. [46] used the Ekphrasis Python library for tokenization. Other studies did not specify which tokenization tool they utilized.

#### 3.6.3. Lemmatization

Six studies lemmatized the text. Lemmatization is an NLP technique used to reduce word inflections into a common root called a lemma [47]. For example, the words “studying”, “studies”, “studied”, and “study” will be reduced to a lemma “study”. This technique allows words with similar meanings to be grouped, reducing the feature space. If lemmatization is not performed, words with the same meaning (e.g., “children” and “child”) will be seen by conventional ML models as unrelated. The authors of [16,40] used NLTK to lemmatize text and [38] used SpaCy for that purpose. Alternatively, [41] opted for stemming instead of lemmatization. Stemming is a similar process that reduces different forms of the word into a single root, which is called stem in this case [47]. Although similar in purpose, stemming is a comparatively crude process that simply drops the ends of words. The words “changing”, “changed” and “change” will be reduced to a stem “chang”. Lemmatization is more accurate than stemming because it conducts a morphological analysis of a word using dictionaries [47]. However, due to its complexity, it tends to be slower than stemming.

Other recurring data preprocessing steps were lowercasing the text and concatenating Reddit post titles and bodies. The studies [45,46] performed spell correction on Reddit posts and [42,50] expanded contractions, e.g., converted “couldn’t” to “could not”.

### 3.7. Feature Engineering

To use ML algorithms, researchers need to extract features from the data. These features then serve as an input to a classifier algorithm. Therefore, the quality of extracted features is one of the factors that significantly affects the predictive performance of the model. Most studies combined techniques to extract different types of features. The researchers primarily focused on extracting features from the textual content of posts. However, several studies also considered statistical metadata, such as the number of posts per user, the frequency of posting, and the number of votes [13,26]. In this section, we outline the most recurring feature extraction techniques.

#### 3.7.1. Term Frequency–Inverse Document Frequency

Term frequency–inverse document frequency (TF–IDF) is the most popular feature extraction technique, used in 14 studies. TF–IDF is used to create a multidimensional vector representation of the entire corpus of preprocessed Reddit data [51]. This technique is based on the idea that in a large body of text, the most recurring words (or terms) have lower semantic importance. Therefore, TF–IDF considers how frequently the words appear in the body of text and assigns different weights to them, giving less-frequent words higher semantic importance. For example, ref. [7] created separate TF–IDF matrices for the title and body of Reddit posts. Tadesse et al. [16] used TF–IDF features with their baseline classifiers that were later used to benchmark the performance of the proposed deep learning model. The TF–IDF features are often used in conjunction with other types of features.

#### 3.7.2. Linguistic Inquiry and Word Count

Linguistic inquiry and word count (LIWC) was used in nine studies to generate linguistic and emotional features from the textual data. LIWC is a powerful lexicon-based tool that helps make inferences about the user’s thoughts, feelings, and personality traits based on the way the person communicates [52]. It has over a hundred dictionaries under different categories that represent an individual’s psychological and social characteristics. Each word in the document is compared against LIWC dictionaries and the number of matches is calculated. For example, if a Reddit post with 100 words is analyzed with LIWC and 10 words relate to negative emotions, the post scores 10% in the negative emotion category. Allen et al. [24] used LIWC to extract features from post titles and bodies. The features were used as inputs to the convolutional neural network (CNN) algorithm. One of the models they developed used features from LIWC’s affect category: negative affect, anger, anxiety, and sadness. They found that a CNN model performed better with LIWC features than with GloVe word embedding vector as an input. The study of [13] used LIWC to conduct a comparative analysis of suicidal and non-suicidal Reddit content. They found that users with suicidal ideations scored significantly higher in the negative emotion, anxiety, and sadness categories and they used personal nouns and present tense more often as compared to average users.

#### 3.7.3. Lexicon-Based Methods

Although not as common as LIWC, there were other lexicon-based feature extraction methods used by the studies. Five studies used NRC (National Research Council Canada) Lexicons to score the Reddit text. Specifically, refs. [38,53] used NRC Word–Emotion Association Lexicon and [45] used NRC Affect Intensity Lexicon to score individual posts in anger, anticipation, disgust, fear, joy, sadness, surprise, and trust categories, whereas [15,49] used NRC Valence, Arousal, and Dominance (VAD) Lexicon. Six other studies used lexicons to obtain sentiment scores for Reddit posts. The sentiment lexicons include AFINN, Senticnet, SentimentDictionaries, and SentiWordNet.

#### 3.7.4. Latent Dirichlet Allocation

Latent Dirichlet allocation (LDA) is another commonly used feature extraction technique. Topic modeling with LDA was used by eight studies to identify hidden topics in Reddit data. LDA is an unsupervised generative probabilistic method used for modeling a body of text [54]. The fundamental idea behind LDA is that each document (a Reddit post in this case) is represented as a mixture of latent topics and each topic is represented as a probabilistic distribution over words. The words with the highest probabilities suggest what that specific topic can be. Matero et al. [15] used LDA to infer 25 topics from r/SuicideWatch posts. Ruiz et al. [53] used LDA to identify 10, 20, and 30 topics from textual data. Ji et al. [44] applied LDA to extract latent topics from posts to represent a person’s suffering, such as negative events and life experiences. However, these studies do not provide lists of discovered topics. Jones et al. [17] used LDA to extract seven topics from all Reddit posts, both suicidal and non-suicidal. The topics include suicide help, social relationship, technology review, human rights, video games, general advice, and services/sales. Then, they demonstrated which topics are frequently discussed by users with different suicide risk levels. They found that users with higher levels of suicide risk focus more on suicide help and social relationship topics.

#### 3.7.5. Statistical Features

Seven studies incorporated statistical features in their suicidality detection model. The authors of [13,15,16] extracted statistical features, such as the number of characters, words, tokens, and sentences per post. Gaur et al. [26] considered the post’s upvotes and downvotes to calculate a controversiality score, based on the idea that equal numbers of upvotes and downvotes indicate that a Reddit post is controversial. Ríssola et al. [46] used the total number of posts per user and the number of subreddits as statistical features to predict suicide risk levels.

#### 3.7.6. Word Embeddings

An alternative to feature engineering is the use of pre-trained word embeddings. Word embedding is an unsupervised learning method used to learn a mapping of each word to a multidimensional vector of real numbers [51]. Similar to TF–IDF, word embedding creates a vector representation of text. However, the main advantage of this approach over TF–IDF is that words with similar semantic meanings (e.g., “frog” and “toad”) have similar vector representations [55]. In contrast, the words “frog” and “toad” would be perceived as different and unrelated in the TF–IDF feature space. Another advantage of using word embedding is that it can generate a fixed-size vector with a smaller number of dimensions [55]. The word embedding techniques are normally used with deep learning algorithms where the resulting vector serves as an input to a deep learning model. The most recurring word embedding technique in the corpus is GloVe, which was utilized by 8 out of 18 studies that applied deep learning algorithms. For example, ref. [56] used GloVe to create a 300-dimensional vector that fed into a hidden layer consisting of CNN, bidirectional recurrent neural network (Bi-RNN), bidirectional long short-term memory (Bi-LSTM), and bidirectional gated recurrent units (Bi-GRU).

Three studies [15,42,50] used bidirectional encoder representations from transformer (BERT) embeddings. BERT is a pre-trained language model that uses a layer of trans-former encoders to produce a sentence or word-level representations of the input text. The pre-trained BERT model can be fine-tuned for a specific task using training data. For instance, ref. [50] fine-tuned the BERT model with training data from CLPsych 2019 Shared Task. Their proposed model—consisting of BERT embedding and Softmax layers—achieved a macro *F*1-score of 0.477 at classifying suicide risk levels. Similarly, ref. [42] developed several suicidal ideation detection models that paired BERT, ALBERT, RoBERTa, and XLNet with a Softmax output layer. They compared the performance of these models with a baseline made of Bi-LSTM and GloVe. The results of the experiments revealed that the RoBERTa-based model had the highest performance, achieving 95.21% accuracy. Matero et al. [15] used dimensionally reduced BERT embeddings with statistical and theoretical lexicon-based features. The authors found that combining BERT embeddings with theoretical features resulted in better performance at predicting the levels of suicide risk. Alternatively, ref. [39] used a transformer-based universal sentence encoder to generate sentence embeddings of Reddit data. The authors found that a fully connected neural network used with a universal sentence encoder outperformed the baseline models built with TF–IDF and Word2Vec and achieved an accuracy of 94.16%.

#### 3.7.7. Dimensionality Reduction

Generating features for a dataset containing long passages of text results in vectors with a high number of dimensions [55]. The resulting vector of features represents the independent variables that are used to predict a target class. In addition to requiring more computational resources, the high dimensionality can lead to poor performance of the model because it might fail to find important signifying patterns in the data [55]. The indicative features might be less prominent among numerous irrelevant features. To tackle this issue, several studies used dimensionality reduction techniques. Aladağ et al. [7] used a one-way analysis of variance F-test to reduce the number of features in TF–IDF vectors, whereas [13,17] used principal component analysis. Shah et al. [41] used a combination of filter and wrapper feature selection methods. The non-negative matrix factorization and chi-squared test methods were used by [15,46], respectively.

### 3.8. Model Development

All the studies in the corpus frame their contributions as building a predictive model that detects suicidal ideations from Reddit data. They tested multiple algorithms with different sets of features and proposed best-performing models. In total, 21 supervised ML algorithms were explored by the researchers. Most studies (18 out of 26 studies) included deep learning techniques. The researchers chose deep learning because, when used in conjunction with word embeddings, the deep-learning-based models can effectively detect suicidal ideations without the need for feature engineering. However, three studies showed that standard ML methods outperform newer deep learning techniques [13,38,49]. Shing et al. [38] attributed the deep learning model’s poorer performance to the small size of training data.

#### 3.8.1. Support Vector Machine

The most recurring algorithm in the corpus was the support vector machine (SVM), which was included in experiments in 19 studies. SVM is a supervised ML algorithm that can be used for classification and regression tasks [47]. Using training data samples, called support vectors, the algorithm constructs an optimal hyperplane that separates samples into two classes. If support vectors are non-linearly separable, SVM applies a technique called the kernel trick to map them to a higher-dimensional space where a hyperplane can be determined [51]. Although SVM is a binary classifier, it can be adapted for multiclass classification, by dividing the original problem into several binary classification subproblems [55]. The author of [7] proposed SVM as the best-performing algorithm for classifying posts into suicidal and non-suicidal categories. Shing et al. [38] found that SVM outperformed logistic regression, XGBoost, and CNN in the multiclass problem where Reddit users were categorized into different levels of suicide risk.

#### 3.8.2. Logistic Regression

The logistic regression (LR) algorithm was included in 11 studies. LR learns the function that models the relationship between independent variables (features) and a dependent variable (a target class) to make a prediction. It estimates the probability of an event occurring—such as a Reddit user being suicidal or non-suicidal—based on a given set of features. The outcome of LR is a probability value between 0 and 1. For a binary classification task, probability lower than 0.5 will predict 0 (negative class) and probability greater than 0.5 will predict 1 (positive class) [57]. LR was the best-performing algorithm in three studies [7,46,58]. Hevia et al. [49] experimentally chose an ensemble model made of SVM and LR as their best model for predicting the different suicide risk levels among Reddit users.

#### 3.8.3. Deep Learning Algorithms

Long short-term memory (LSTM) and convolutional neural network (CNN) are the two most popular deep learning methods in the corpus and appear in 13 and 12 studies, respectively. CNN is an artificial neural network that was originally intended for computer vision, but it later found its application for text classification [59]. LSTM is a popular variation of recurrent neural networks (RNNs), which addresses the issues of gradient vanishing or explosion, often experienced with standard RNN architectures [51]. The study of [44] applied CNN over the word embedding to create feature maps and used a max-pooling layer over the features. Three studies [11,16,40] proposed an ensemble model that combines LSTM and CNN. Renjith et al. [40] proposed an LSTM-Attention-CNN model with a 300-dimensional Word2Vec embedding acting as an input layer. Similarly, ref. [16] proposed an LSTM-CNN model that also applied the Word2Vec technique at a word embedding layer. In contrast, ref. [11] used a ConceptNet as the embedding technique with their proposed CNN+LSTM model. Table 2 shows a summary of features and algorithms used by the reviewed studies.

### 3.9. Model Validation

Once the predictive model is trained, the performance of the model is evaluated. The most common evaluation metrics include accuracy, precision, recall, and *F*1-score. However, two studies also calculated the area under the curve (AUC) metric [11,26]. The last column in Table 2 summarizes the metrics used in each of the studies.

For suicidality detection task, true positive (TP) represents the number of posts that were correctly classified as suicidal. True negative (TN) represents the number of posts that were correctly classified as non-suicidal. False positive (FP), also known as Type I error, represents the number of non-suicidal posts that were misclassified as suicidal. False negative (FN), also known as Type II error, represents the number of suicidal posts that were misclassified as non-suicidal.

Accuracy measures the overall portion of correct predictions [51]. It is a ratio of all correctly classified posts to the total number of posts:

(1) $$Accuracy=\frac{TP+TN}{TP+FP+TN+FN}$$

Precision is a ratio of correctly classified suicidal posts to the total number of posts classified as suicidal (both correctly and incorrectly) [51]:

(2) $$Precision=\frac{TP}{TP+FP}$$

Recall, also called sensitivity or true-positive rate, is the ratio of correctly classified suicidal posts to the total number of suicidal posts, including both correctly classified posts and posts that should have been classified as suicidal [51]:

(3) $$Recall=\frac{TP}{TP+FN}$$

This metric is especially useful for selecting the best model where there is a high cost of false-negative predictions [61]. In the suicidal ideation detection model, false positives are more tolerable than false negatives [62]. In other words, it is better to raise a false alarm by incorrectly predicting someone as suicidal than to miss someone who is indeed at risk of suicide.

*F*1-score is the harmonic mean of precision and recall:

(4) $$F1=2\times \frac{Precision\times Recall}{Precision+Recall}$$

For multiclass classification problems, the macro-averaged *F*1-score can be determined by calculating individual *F*1-scores for each class and finding their unweighted mean.

The receiver operating characteristic curve is a graph that plots the true-positive rate (Equation (3)) against the false-positive rate (Equation (5)) at different classification thresholds [55]. It provides a graphical representation of the classifier’s performance and a larger area under the curve indicates better performance.

(5) $$False\;Positive\;Rate=\frac{FP}{FP+TN}$$

## 4. Discussion

### 4.1. Limitations

Despite substantial progress and success in detecting suicidal ideations, the current research has limitations, the largest being a lack of data. The researchers typically approached the suicidality detection problem by applying supervised ML techniques, which demand a sufficient amount of annotated data to yield good results. While extracting posts from Reddit might not present a challenge, labeling those posts does. The process of creating an annotated dataset is time consuming as it requires researchers to go through every post and label them [38]. For instance, in the dataset created by [7], only 785 out of 508,398 posts were annotated, which makes up only 0.15% of all extracted posts. Correspondingly, only 8.39% of the UMD Reddit Suicidality Dataset is annotated.

The second limitation that pertains to the dataset is annotation bias. As described earlier, the researchers either enlist the help of domain experts to annotate data or outsource the annotation task to crowdsourcing. Although with the latter approach, researchers manage to get a bigger section of the dataset annotated, the resulting annotations contain bias since the crowdsource workers do not possess knowledge from the mental health domain [38]. When compared to expert annotators, it was shown that crowdsource workers err on the side of caution, labeling more non-suicidal posts as suicidal. The model trained with such data is bound to produce more false positives.

There is a trend where more researchers are opting for deep learning techniques for suicidal ideation detection. The deep learning algorithms are usually paired with embedding methods. While the use of pre-trained embedding models does not require feature engineering and can result in high performance, it becomes challenging to infer the decision rules that were determined by the classifiers to make the predictions [17]. This can become an obstacle for professionals who would like to know what signs to search for in an individual’s online posts that may indicate high suicide risk.

Although Reddit is deemed a valuable source of data due to reasons discussed in the earlier section, there are a few limitations associated with its use. The limitation that several studies specified is the absence of information on Reddit users’ health outcomes [7,38,45]. In other words, it is unknown whether an individual who exhibited suicidal ideations on Reddit attempted or died by suicide after posting on the forum. Without known outcomes, it becomes challenging to assess the clinical validity of models built with Reddit data [38]. It is a crucial consideration, as suicidal ideation detection models built with Reddit data should be primarily seen as systems predicting suicidal ideations, not potential suicide attempts [7]. It is unlike predictive models trained with data from suicide notes and electronic health records, where it is clear if the person indeed committed suicide.

The second limitation of using Reddit as a data source arises from the linguistic characteristics of language used on forums. The Reddit users use an informal style of language, containing slang and abbreviations. It can present a challenge when researchers utilize medical knowledge bases during the feature extraction process. To address this limitation, the authors of [26] used two medical entity normalization lexicons to map informal terms and phrases within Reddit data to formal medical concepts defined in knowledge bases.

Since the research in this domain involves human subjects, concerns over ethics and data privacy exist. Despite anonymity being one of the key characteristics of Reddit, the researchers should still pay attention to users’ data privacy due to the sensitive nature of mental health topics. While Reddit does not require users to provide any identifying information, the users can still supply their personal details in their usernames, profiles, or even post content. For this reason, the creators of the UMD Reddit Suicidality Dataset took additional precautions and removed all personal information using named-entity recognition tools [38].

### 4.2. Future Directions

The current state of research predominantly focuses on detecting suicidal ideation in the textual data. The direction that the research can take in the future is to build models that aim to understand why forum users have suicidal ideation [10]. In other words, the focus of the research can advance from simply detecting the presence of cues to identifying the causes of suicidality. This could be done by incorporating existing suicide- and mental-health-related knowledge bases into predictive models [26]. This would require further interdisciplinary integration of ML and psychology.

Another direction the research can take is addressing intervention. The researchers can apply natural language generation techniques to automatically create a response to the users in distress [27]. The current conversational counseling practices used by clinicians can be studied to develop models that can become the first line in suicide intervention [10]. Such a model can become an element of social media sites that would initiate a conversation with a user exhibiting suicidal ideations to encourage the person to seek professional help.

The researchers can explore solutions for managing the lack of annotated data in future studies. One of the approaches for collecting data containing suicidal ideations can be keyword-based web-crawling techniques. This approach is prevalent in suicidality detection based on Twitter data. Nevertheless, it is potentially applicable for extracting suicidal data from Reddit. The forthcoming studies can improve the effectiveness of keyword-based web-crawling methods by incorporating domain knowledge when designing suicide-signifying search terms. For example, having used knowledge bases and suicide ontology, ref. [26] developed a lexicon for each category of suicide risk and made it publicly available.

Another potential method for sourcing annotations is administering questionnaires to screen study participants for suicidal ideations. It would imply surveying the participants to confirm their mental health status and securing their consent to collect their Reddit data. Similar research domains employed this approach. For example, ref. [63] compared the Twitter data of 135 participants with their responses to suicide risk screening questionnaires. Similarly, ref. [64] used a questionnaire to assess 166 participants’ depression levels and compared responses to their Instagram profiles. It is a viable approach, as the individuals confirm whether they exhibit target mental health symptomatology. However, it is worth noting that it can potentially raise issues of study complexity and participant privacy.

Future research can apply transfer learning techniques to address the lack of large, annotated datasets. The transfer learning approach leverages language models pre-trained with general textual data outside the specific domain. It allows researchers to fine tune the pre-trained models with small, task-specific training datasets. Although only a few studies in the corpus employed transfer learning, their models achieved high accuracy, often outperforming more conventional methods. This approach is valuable for suicidal ideation detection, where the challenge of obtaining reliable annotations restricts training dataset size [17].

The studies can investigate feature selection techniques based on matrix factorization to tackle the high dimensionality issue for input data. These techniques can potentially reduce data redundancy, lower computation cost, and, as a result, help build better predictive models. Such matrix-factorization-based feature selection techniques have been shown to be effective in other health-research domains. For example, the researchers applied modified matrix-factorization-based feature selection methods to determine two genes that can predict the cell’s response to cancer treatment [65]. Further, these techniques were applied to determine the clinical biomarkers that predict health outcomes in COVID-19 patients [66]. Although the nature of the data in these domains is different from textual Reddit data, these techniques can be examined in future research, as a similar non-negative matrix factorization dimensionality reduction method was used by one of the included studies [46].

In the future, the suicidal ideation detection tool can be integrated into existing health-care IT systems. It would be able to assist health-care providers by alerting them when a patient’s mental health worsens, calling them to attend to a patient. Further, such a tool can supplement traditional suicide-risk screening methods, such as questionnaires and interviews, allowing for the identification of suicidal people outside the clinical setting prior to their contact with health-care providers.

## 5. Conclusions

This paper reviewed the current state of the art in research on suicidal ideation detection on Reddit. We started by providing background on suicide, presented the challenges hindering suicide prevention, described motivating factors for search, and outlined the rationale of using Reddit as the data source. Next, we discussed the methods used in the domain for data collection, data annotation, data preprocessing, feature engineering, model development, and evaluation. Our findings revealed that most of the studies approach suicidal ideation detection as a classification problem by applying machine learning and deep learning techniques. We explored common sources of data, reviewed annotation methods, and provided examples of common features and algorithms. Lastly, we discussed the current limitations and possible future directions of the research.

## Figures and Tables

**Table 1:** Data source: (**a**) post level and (**b**) user level.

**(a)**				
**Dataset**	**Total Size**	**Annotated Subset**	**Annotation Source**	**Classes**
Aladağ et al., 2018 [7]	508,398 posts	785 posts	Experts	Suicidal, non-suicidal
Ji et al., 2018 [13]	3549 suicidal posts 3652 non-suicidal posts	NA	Community affiliation	Suicidal, non-suicidal
Yao et al., 2020 [25]	NA	500 r/Opiates posts 500 r/SuicideWatch posts	Crowdsourcing	Opioid addiction, no opioid addiction, suicide risk, no suicide risk
Reddit SuicideWatch and Mental Health Collection by Ji et al., 2021 [44]	54,412 posts	NA	Community affiliation	r/Depression, r/SuicideWatch, r/Anxiety, r/Offmychest, r/Bipolar
Nikhileswar et al., 2021 [39]	116,037 suicidal posts 116,037 non-suicidal posts	NA	Community affiliation	Suicidal, non-suicidal
**(b)**				
**Dataset**	**Total Size**	**Annotated Subset**	**Annotation Source**	**Classes**
UMD Reddit Suicidality Dataset v2 by Shing et al., 2018 [38]	11,129 r/SuicideWatch users 11,129 non-r/SuicideWatch users	866 r/SuicideWatch users 866 non-r/SuicideWatch users	Crowdsourcing, experts	No risk, low risk, moderate risk, severe risk
Reddit C-SSRS Suicide Dataset by Gaur et al., 2019 [26]	NA	500 users (15,755 posts)	Experts	Indicator, ideation, behavior, attempt, supportive
Reddit C-SSRS Suicide Dataset v2 by Gaur et al., 2021 [11]	NA	448 users (7327 posts)	Experts	Ideation, behavior, attempt, supportive

**Table 2:** Summary of machine learning and natural language processing techniques.

Study	Feature Extraction Techniques	Machine Learning Algorithms	Embedding Techniques	Deep Learning Algorithms	Best Performing Model	Metric and Result
Shing et al., 2018 [38]	BOW, Empath, Readability Index, Syntactic features, LDA, LIWC, NRC Lexicon, mentalDisLex (Mental Disease Lexicon)	SVM, LR, XGBoost	SkipGram	CNN	SVM	Macro *F*1 = 0.460
Aladağ et al., 2018 [7]	TF–IDF, LIWC, Sentiment	ZeroR, LR, RF, SVM	NA	NA	LR, SVM	Accuracy = 0.920 Accuracy = 0.920
Ji et al., 2018 [13]	Statistics, Part of Speech Tags, LIWC, TF–IDF, LDA	SVM, RF, Gradient Boost Decision Tree, XGBoost,	Word2Vec	MLFFNN, LSTM	XGBoost	Accuracy = 0.957
Allen et al., 2019 [24]	LIWC	NA	GloVe	CNN	CNN used with LIWC	Macro *F*1 = 0.500
Ambalavanan et al., 2019 [50]	NA	NA	BERT	LSTM	BERT-Softmax	Macro *F*1 = 0.477
Bitew et al., 2019 [58]	TF–IDF, DeepMoji pre-trained model,	LR, SVM,	NA	NA	LR	Macro *F*1 = 0.445
Chen et al., 2019 [48]	Sentiments, LIWC, EMPATH, TF–IDF, Statistics	SVM	NA	NA	SVM	Macro *F*1 = 0.380
Gaur et al., 2019 [26]	Sentiments with AFINN, TF–IDF, Statistics, Syntactic	SVM, RF	ConceptNet	FFNN, CNN	CNN	Graded Recall = 0.600
González Hevia et al., 2019 [49]	TF–IDF, NRC VAD Lexicon	SVM, LR	Multilingual Word Embedding, Doc2Vec	RNN	SVM-LR	Macro *F*1 = 0.320
Iserman et al., 2019 [45]	Sentiments with AFINN, Hu & Liu, General Inquirer, labMT, LIWC, Lusi, Moral Foundations, Netspeak, NRC Affect Intensity Lexicon, Senticnet, SentimentDictionaries, SentiWordNet, Slangsd, Vader, Whissell, Age&Gender, PERMA	LR, RF, DT	NA	NA	DT	Macro *F*1 = 0.402
Matero et al., 2019 [15]	Affect & Intensity Lexicon, NRC VAD Lexicon, Age&Gender Lexicon, Big-5 Personality Lexicon, Anxiety, Anger & Depression Lexicon, LDA, Statistics	LR	BERT	LSTM	LSTM-Attention	Macro *F*1 = 0.500
Mohammadi et al., 2019 [56]	NA	SVM	GloVe, Embeddings from Language Model	CNN, RNN, LSTM, GRU,	Ensemble model consisting of CNN, Bi-RNN, Bi-LSTM, Bi-GRU and SVM	Macro *F*1 = 0.481
Morales et al., 2019 [60]	BOW, TF–IDF, LDA, POS, Named-Entity Recognition, IBM Watson Personality Insights API, IBM Watson Tone Analyzer	RF, NB, KNN, SVM	SkipGram, FastText	CNN, LSTM, NeuNetS	CNN	Macro *F*1 = 0.310
Ríssola et al., 2019 [46]	TF–IDF, LIWC, Statistics	LR, SVM, RF	GloVe	N/A	LR	Macro *F*1 = 0.311
Ruiz et al., 2019 [53]	Clinical Text Analysis and Knowledge Extraction System, Social Determinant of Health, NRC Word-Emotion Association Lexicon, Readability Index, Semantic Role Labeling, Sentiments, LDA, Empathy	NB, GB, RF, SVM,	Doc2Vec	CNN, LSTM,	Ensemble model consisting of NB, SVM, GB	Macro *F*1 = 0.379
Jones et al., 2019 [17]	Suicide Risk Factor Lexicon, LDA	RF, LR, SVM	FLAIR, GloVe	N/A	RF	*F*1 = 0.920
Tadesse et al., 2019 [16]	Statistics, TF–IDF, BOW,	RF, SVM, NB, XGBoost	Word2Vec	LSTM, CNN	LSTM-CNN	Accuracy = 0.938
Shah et al., 2020 [41]	TF–IDF, N-Gram, LIWC	NB, SVM, KNN, RF	NA	N/A	NB	Accuracy = 0.736
Yao et al., 2020 [25]	TF–IDF	LR, RF, SVM,	GloVe, FastText	RNN, CNN	CNN	*F*1 = 0.966
Haque et al., 2020 [42]	NA	NA	Glove, BERT	LSTM	BERT with Softmax Layer	Accuracy = 0.952
Kumar et al., 2021 [43]	NA	NB, LR, SVM	GloVe	LSTM, GRU	Bi-GRU with Multiplicative Attention	Micro *F*1 = 0.300
Rabani et al., 2021 [30]	TF–IDF, BOW, Statistics, LDA, POS	NB, DT, LR, SVM,	NA	N/A	DT	*F*1 = 0.980
Gaur et al., 2021 [11]	NA	NA	ConceptNet	CNN, LSTM	CNN-LSTM	AUC = 0.640
Ji et al., 2021 [44]	Sentiments, LIWC, LDA	NA	GloVe, FastText	CNN, LSTM, Structured Self-Attentive Sentence Embedding, Relation Network	Relation Network	*F*1 = 0.545
Nikhileswar et al., 2021 [39]	TF–IDF, BOW	XGBoost, SVM	Universal Sentence Encoder, Word2Vec,	LSTM, CNN, FCNN	FCNN used with Universal Sentence Encoder	Accuracy = 0.942
Renjith et al., 2021 [40]	TF–IDF	SVM	Word2Vec	LSTM, CNN	LSTM-Attention-CNN	Accuracy = 0.903

## Data Availability

Not applicable.

## Abbreviations

The following abbreviations are used in this manuscript.

BERT	Bidirectional Encoder Representations from Transformers
BOW	Bag of Words
CNN	Convolutional Neural Network
DT	Decision Tree
FCNN	Fully Connected Neural Network
FFNN	Feedforward Neural Network
GRU	Gated Recurrent Units
KNN	K-Nearest Neighbors
LDA	Latent Dirichlet Allocation
LIWC	Linguistic Inquiry and Word Count
LR	Logistic Regression
LSTM	Long Short-Term Memory
MLFFNN	Multilayer Feed Forward Neural Net
NB	Naïve Bayes
NRC	National Research Council Canada
RF	Random Forest
RNN	Recurrent Neural Network
SVM	Support Vector Machines
TF–IDF	Term Frequency–Inverse Document Frequency
XGBoost	Extreme Gradient Boosting
